# An Audit of Clinical Practice in a Single Centre in Kuwait: Management of Children on Continuous Subcutaneous Insulin Infusion and Cardiovascular Risk Factors Screening

**DOI:** 10.2174/1874192401711010019

**Published:** 2017-02-28

**Authors:** Dina Omar, Hala Alsanae, Mona Al Khawari, Majedah Abdulrasoul, Zahraa Rahme, Faisal Al Refaei, Kazem Behbehani, Azza Shaltout

**Affiliations:** Dasman Diabetes Institute, Al-Amiri Hospital, Faculty of Medicine, University of Kuwait, Kuwait, Kuwait

**Keywords:** Audit, Continuous subcutaneous insulin infusion, Diabetes pump therapy, Hypoglycaemia, Diabetes Control and Complications Trial (DCCT), Glycated haemoglobin (HbA1c), Type 1 diabetes mellitus

## Abstract

**Objectives:**

To audit the current clinical practice of continuous subcutaneous insulin infusion (CSII) for the treatment of type 1 diabetes mellitus (T1D) in children and adolescents attending a single centre in Kuwait.

**Methods:**

A one year retrospective audit was performed in children and adolescents with T1D on CSII, who attended the paediatric diabetes clinic, Dasman Diabetes Institute during 2012. The primary outcome measure was glycaemic control as evidenced by glycated haemoglobin (HbA1c) level and the secondary outcome measures were the frequency of monitoring of the risk for microvascular complications and occurrence of acute complications and adverse events.

**Results:**

58 children and adolescents (mean age ± SD: 12.6 ± 4.1 years) were included. Mean HbA1c at baseline was 8.8% (72.7 mmol/mol) and 8.9% (73.8 mmol/mol) at the end of a 12 months observation period. Children with poor control (HbA1c >9.5% (80 mmol/mol) had a significant 1.4% reduction in HbA1c compared with the overall reduction of 0.1% (p=0.7). Rate of screening for cardiovascular risk factors and for long term complications were well documented. However, there was underreporting of acute complications such as severe hypoglycaemia and diabetic ketoacidosis. Only 1.7% of patients discontinued the pump.

**Conclusion:**

There was no significant change in HbA1c values at the end of 12 months follow up. However, HbA1c values in poorly controlled children improved. CSII requires care by skilled health professionals as well as education and selection of motivated parents and children.

## INTRODUCTION

Several studies have established the importance of achieving good glycaemic control in children with type 1 diabetes (T1D) [1]. Results from the Diabetes Control and Complications Trial (DCCT) and the Epidemiology of Diabetes Interventions and Complications (EDIC) [2] follow up study of the DCCT cohort have demonstrated that most people with T1D should be treated intensively to achieve haemoglobin A1c (HbA1c) levels as close to normal as possible and as early as possible to prevent and/or delay the late micro- and macrovascular complications of the disease [3]. Recommendations from the DCCT include that adolescents with T1D should be treated intensively with multiple daily injections (MDI) of insulin or continuous subcutaneous insulin infusion (CSII) [4]. CSII or insulin pump therapy, helps to achieve better metabolic control as it simulates the function of the insulin-secreting islet cells more closely than MDI [5, 6]. Moreover, important advantages of CSII include decreased variability in insulin absorption, and therefore more predictable and reproducible outcome [7].

Although CSII is not currently an economically appealing option for patients with T1D in some countries like the United States [8], it was found to be a cost-effective option in other countries like Bulgaria [9]. Indeed, considering the improvement in quality of life and the potential risk reduction of long term complications, CSII may prove to be a cost-effective alternative to MDI [10]. One study demonstrated that HbA1c one year post CSII was not significantly different from baseline but CSII use led to a significant reduction in the incidence of severe hypoglycaemia SH [11].

“Clinical audit” is process that seeks to improve patient care and clinical outcome through a systematic review of care based on defined criteria, and the implementation of change. The key component of clinical audit is that performance is reviewed to ensure that it provides a framework to enable improvements to be made [12]. In 2008, the National Institute for Health and Clinical Excellence (NICE), issued a technology appraisal guidance to measure current practice in CSII for the treatment of diabetes mellitus and provided 8 audit criteria to be followed [12].

We undertook an audit to evaluate the efficacy and safety of CSII in a clinic population attending the Dasman Diabetes Institute (DDI), Kuwait. We assesed adherence to well established clinical guidelines in a well-defined cohort of children and adolescents.

## METHODS

DDI is a non-profit organization that was established by the Kuwait Foundation for the Advancement of Sciences (KFAS) in 2006. The mission of the institute is to prevent, control and mitigate the impact of diabetes and related conditions in Kuwait through effective programs of research, training, education, and health promotion and thereby improve quality of life in the population. Children with poorly controlled diabetes are referred from all over Kuwait to the centre to optimize their management.

CSII has been available for use in clinical practice in Kuwait for several years. A map of the locations of 11 healthcare centres providing insulin pump therapy for adults and children is shown in Fig. (**1**). A total of 366 pumps were installed for children and adults all over Kuwait since 2009 until the first quarter of 2013.

Standards of audit were created concerning CSII: the primary outcome measure was the difference between HbA1c at baseline (pre-pump) and at the end of 1 year audit period. Secondary outcome measures were to determine the proportion of children who had their blood pressure (BP) measured, urine checked for microalbuminuria (MA), lipid levels measured, or fundus examination according to the International Society of Pediatric and Adolescent Diabetes (ISPAD) consensus guidelines [17].

Inclusion criteria were: being on CSII for >30 days, at least 2 recordings of HbA1c levels, and attending outpatient department appointments at least 3 times during the study period. Before starting CSII, candidate patients had to attend a 4-6 day structured educational program, delivered by a specialized nurse diabetes educator and registered dietitian.

### Data Collection

The electronic health records were retrospectively reviewed. Collected data included age at initiation of CSII, gender, duration of diabetes prior to start of CSII, anthropometric measurements, BP, pre-pump HbA1c and HbA1c at the end of 12 months. Other measurements, such as fundus screening, lipid profile and urinary MA based on ISPAD guidelines were also included. Additionally, 3 audit fields were completed for each child – the number of admission for diabetic ketoacidosis (DKA), severe hypoglycaemic episodes and of adverse events due to CSII during the audit year.

### Definitions

Recent American Diabetes Association (ADA) guidelines have set the target HbA1c for children with diabetes at a level <7.5% (58.5 mmol/mol) [13]. Poor control was defined as a HbA1c of >9.5% (80 mmol/L) which is above the 3^rd^ quartile for results of a large diabetic population as part of a national audit in England [14]. DKA was defined as hyperglycaemia of >11 mmol/L, venous pH <7.3 or bicarbonate <15 mmol/L, ketonaemia and ketonuria [15]. Severe hypoglycaemia was defined as altered mental status in children who cannot assist in their care, in coma or convulsions and may require parenteral therapy [16].

### Biochemical Analysis

HbA1c was measured by high-performance liquid chromatography. MA was measured by 2 techniques, nephelometry and turbidimetry. Normal values for HbA1c and MA were 4-6% (20.2- 42.1 mmol/mol) and 1-25 mg/l, respectively.

### Statistical Methods

All statistical analyses were carried out using Statistical Analysis System (SAS). For statistical tests we used non-parametric methods. We used analyses of covariance to compare values at baseline with those at 12 months between the study arms. The Student t test was used for comparison between age groups. A two-tailed p < 0.05 was considered significant.

## RESULTS

The retrospective case-note audit identified 58 children out of 415 (13.9%) who were on CSII and attending the Ambulatory Paediatric Diabetes service and fulfilling the criteria for inclusion. The 12 month electronic chart review period started from January 1, 2012 until December 31, 2012. The mean age (± SD) was 12.7 ± 4.1 (median 12.9 years, range 4.4-19.0) with a male:female ratio of 0.9. Seventy five per cent were Kuwaiti children. Only 3 patients (5.2%) were <6 years of age, 23 (39.7%) were aged 6 and 12 years and 32 patients (55.1%) were >12 years old. The mean duration of T1D was 4.6 ± 4.0 years. The mean duration of CSII at the beginning of the study period was 18.4 months.

### Indications for CSII

The majority (50.0%) installed the pump, for better quality of life, 17 patients (29.3%) for improvement of hypoglycaemia, 7 (12.1%) because of recurrent DKA, 3 (5.2%) for recurrent severe hypoglycaemic attacks and 2 (3.4%) because of needle phobia.

Overall, pre-pump HbA1c value was 8.8 (72.7 mmol/mol) ± 1.20%, and became 8.9 (73.8 mmol/mol) ± 1.2% at the end of 12 months (p=0.7) (Table **1**). The median HbA1c was 8.6% (70 mmol/mol) and mean HbA1c was 8.9% (73.8 mmol/mol), 8.8% (72.7 mmol/mol) 8.6% (70.5 mmol/mol) and 8.9% (73.9 mmol/mol) at 3, 6, 9 and 12 months, respectively. Age groups <6, years 6-12 and >12 years had a baseline mean HbA1c of 8.3 (67.2 mmol/mol) ± 0.6%, 8.7 (71.6 mmol/mol) ± 1.2% and 9.0 (74.9 mmol/mol) ± 1.3%, respectively. Corresponding values at the end of the observation period were 8.4 (68.3 mmol/mol) ± 0.4, 8.1(65.0 mmol/mol) ± 0.8% and 9.5 (80 mmol/mol) ± 1.6% (Table **1**).

The 6-12 years old age group was the only group that had decreased HbA1c with an absolute reduction of 0.6% from baseline to reach 8.1 (65.0 mmol/mol) ± 0.8%, although this was not significant (p=0.08) (Table **1**). The adolescent group had the highest HbA1c throughout the follow up period.

**Fig. (2):**
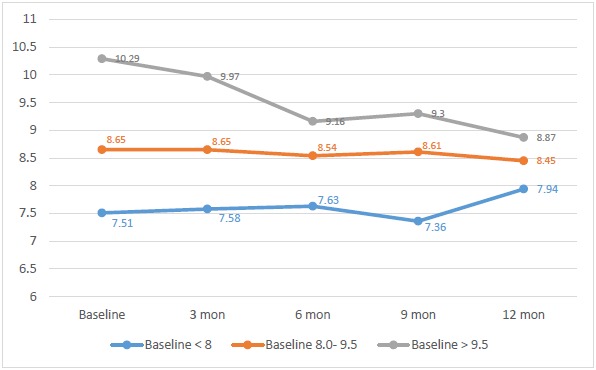
Serial HbA1c at baseline, 3, 6, 9, and 12 months of children and adolescents on Continuous Subcutaneous Insulin Infusion (CSII) according to baseline control defined by glycated haemoglobin (HbA1c) level.

CSII was shown to be more effective, in terms of HbA1c reduction, in those with poor glycaemic control. (Fig. **2**) Poorly controlled children and adolescents, with baseline HbA1c of > 9.5% (80.3 mmol/mol), achieved a significant (p= 0.004) improvement with 1.4% reduction at the end of the one year audit (Table **2**).

Those with baseline HbA1c <8.0% (63.9 mmol/mol) had an increment of 0.4% by the end of the observation period, an increase which was not significant. On the other hand, those with baseline HbA1c of 8.0-9.5% (63.9 - 80 mmol/mol) had a non-statistically significant reduction by 0.2%. Table **3** shows the change in HbA1c and absolute reduction or increase in HbA1c; 46.6% achieved a reduction *vs* 34.4% who showed an increase in HbA1c.

**Fig. (3):**
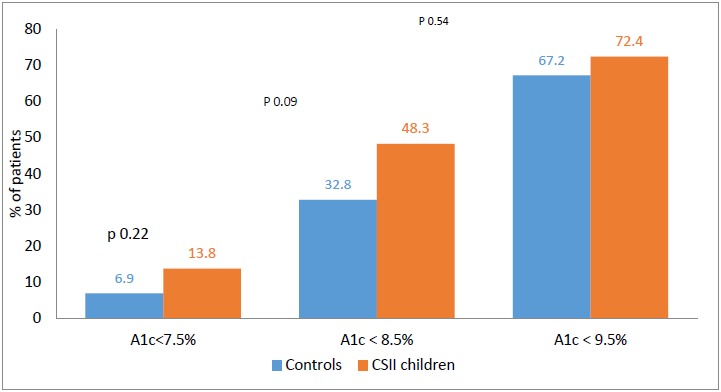
Cumulative percentage of HbA1c in 58 patients on Continuous Subcutaneous Insulin Infusion (CSII) *vs* controls (Multiple Daily Injections).

To compare with the study population, 58 children on MDI were matched for age, baseline HbA1c and BMI during the same period. Mean HbA1c of children on CSII, 8.9 (72.7 mmol/mol) ± 1.7%, was lower than the mean HbA1c of 9.2% (77.0 mmol/mol) ± 1.3 for the control group, (p=0.29). Children on CSII were more likely to achieve optimal control, HbA1c <7.5 (58 mmol/mol) 13.8 *vs* 6.9% (p= 0.22) and 48.3 *vs* 32.8% on MDI had HbA1c <8.5% (p= 0.09). Moreover, 72.4% of the 58 children on CSII had an HbA1c level <9.5 *vs* 67.2% for the control group (p=0.54) (Fig. **3**). However, these differences were not significant.

Most children above the age of 10 years (82.5%) had their lipid profile checked once during the study period. Target levels of lipid in children with T1D were total cholesterol level < 5.2 mmol/l, high density lipoprotein cholesterol (HDL-C) >1 mmol/l, low density lipoprotein cholesterol (LDL-C) < 2.6, and triglyceride (TG) level < 1.7 mmol/l [17]. Overall, 21.7, 4.4, 50.0 and 6.5% had levels outside target range for total cholesterol, HDL-C, LDL-C and TG, respectively.

To audit the adherence to accepted guidelines, the global IDF/ISPAD guidelines was adopted, which recommend screening for fasting blood lipids in children with diabetes at the age of 10 years [17]. Indication for testing for MA was age >10 years old or T1D duration of >5 years.

Screening for other parameters was well documented. BP was checked in the clinic for 41 patients (70.7%) at least once during the year (2012); fundoscopy screening for retinopathy was performed for 94.8% of patients, and MA was tested in all patients for whom the test was indicated. T1D-related acute complications such as SH, emergency room visits for DKA and technical problems with the pump were not routinely documented in the electronic health records. This information was documented in only 10.3% of the records, which made it difficult to draw conclusions about their frequency.

Only 1 patient (1.7%) discontinued pump use during the 12 month study period due to recurrent blocking of the tubing.

## DISCUSSION

This retrospective study aimed to audit the clinical practice of using CSII for the management of children and adolescents with T1D in a single tertiary centre in Kuwait. To date, this is the first cycle of study that audits the use of CSII in Kuwait. Overall, HbA1c had a marginal non-statistically significant increase of HbA1c from 8.8% (72.7 mmol/mol) to 8.9% (73.8 mmol/mol).When serial HbA1c of children and adolescents with T1D on CSII, were compared with a matched control on MDI, during the same time period, the proportion of children and adolescents achieving good glycaemic control was higher with CSII than those on MDI (13.8 *vs* 6.9%). The lack of statistical significance may be related to the small sample size (*n*)**.** An important finding in this study, was that, children and adolescents with high baseline HbA1c, were able to achieve a significant reduction in HbA1c on CSII. Screening for long-term complications of T1D was well-documented, but recording of adverse events was not.

The overall mean HbA1c of the study cohort, HbA1c 8.9% (73.8 mmol/mol), at the end of 12 month period was lower than the mean HbA1c of a control group on MDI, 9.2% (77.0 mmol/mol) ± 1.3. Furthermore, 13.8% of children and adolescents achieving a target HbA1c <7.5%, using CSII *vs* 6.9% of children on MDI. The fact that it did not reach statistical significance may be related to the small *n*.

While glycaemic control improved by the end of the 12 month study period in the age group 6-12 year old children, it deteriorated in the younger and older age groups. Other studies have also shown initial improvement in the level of HbA1c with later deterioration relative to pre-CSII [18, 19]. However, when children with poorly controlled diabetes (defined as HbA1c of >9.5% (80 mmol/mol)) were considered, a significant reduction was evident at the end of 12 months (p=0.02).

Sustainability of HbA1c improvement is not always consistent through the different age groups in the published literature. Children with T1D switching from MDI to CSII took 4 years of follow up in a study to show a significant reduction of baseline HbA1c [20]. A period of 12 months may be too short to show the benefits of CSII [21]. Although CSII is the gold standard for treatment of children with T1D below the age of 5 years [22] the number of children in this age group was small in our study.

The recent T1D Exchange study, collected from clinic registry from individuals with T1D regardless of their treatment regimen, revealed that during childhood, the mean HbA1c levels decreased from 8.3% (67.2 mmol/mol) in 2-4 year-olds to 8.1% (65.0 mmol/L) at 7 years of age followed by an increase to 9.2% (77.0 mmol/mol) at 19 years old. Similarly, the goal for HbA1c of <7.5% (58 mmol/mol), set by the ADA and ISPAD, was achieved by only a small percentage of children and adolescents (17-23%) [29]. Adolescents in the registry achieved a mean HbA1c of 9.0% compared with the 9.5% registered by the same age group during the DCCT [3]. Furthermore Scottish children with T1D had a mean HbA1c of 9.2 ± 1.5 and only 9.7% achieved the target HbA1c <7.5% [23], an experience that is similar to ours. On the other hand, children using insulin pumps tended to have lower HbA1c values of 8.0 ± 0.9, 8.2 ± 1.2 and 8.7 ± 1.5% in 2-5, 6-12 and 13-17 years old, respectively [24] which are lower than what was seen in the present study.

Adherence to ISPAD guidelines was encouraging but we were disappointed by the lack of documentation of acute complications of T1D and the reporting of adverse effects of pump use. Our discontinuation rate (1.7%) was comparable to but less than what has been found by Justen et al. (5.6%) after a mean follow up of 19 months [25], as opposed to other studies that documented discontinuation rate between 18-32% [26-28].

Reaching a near-normal glycaemia control in children and adolescents can be challenging because of inconsistent eating habits and variable activity levels [1]. Clearly, advances in diabetes management over the last two decades have been less successful in overcoming the special challenges in managing teenagers than adults with T1D [26]. The hallmark strategy to improve outcomes is to engage patients, especially adolescents, encourage frequent blood glucose monitoring, have greater access to newer technology such as continuous glucose sensors, as well as increase contact time with the multidisciplinary team to have significant impacts on their glycaemic control. In addition to intensifying patient education and motivation, continuous glucose monitoring (CGM) can serve as an effective way to improve glycaemic control in patients with CSII and was associated with better control of diabetes [25]. In the current study, however, CGM was not used.

This study has several limitations. First, the audit examined retrospective data, which are subject to bias by missing information and absence of data on confounding factors. Second, the study did not take into consideration the duration of T1D or duration of CSII use. Thirdly, the number of participants was small and the duration of 1 year may have not been sufficient to comprehensively evaluate glycaemic control.

Clinical audit, forms part of clinical governance which aims to ensure that patients receive the best quality of care. For the audit to be effective, it should be repeated to find out whether [22] improvement took place. The importance of audit in the healthcare sector needs to be appreciated by the relevant authorities.

## CONCLUSION

This report highlights several aspects of our clinical practice that require improvement. Children and adolescents on CSII were more likely to achieve better glycaemic control compared to those on MDI. The lack of statistical significance may be related to the small n. However, adolescents had more often suboptimal glycaemic control. Healthcare professionals adhered to ISPAD guidelines in screening for hypertension as well as diabetic microvascular complications of retinopathy and nephropathy. CSII therapy effectiveness at reducing major complications like DKA and SH could not be demonstrated. A next audit with a larger *n* and a longer duration is to be considered to allow for statistical significance and be more reliable for clinical decision making.

Recommendations: Although the number of participants were small, there is a considerable room for improvement. A second cycle of audit is planned to allow measurements of progress in meeting standards and input into a local quality improvement.

## Figures and Tables

**Fig. (1):**
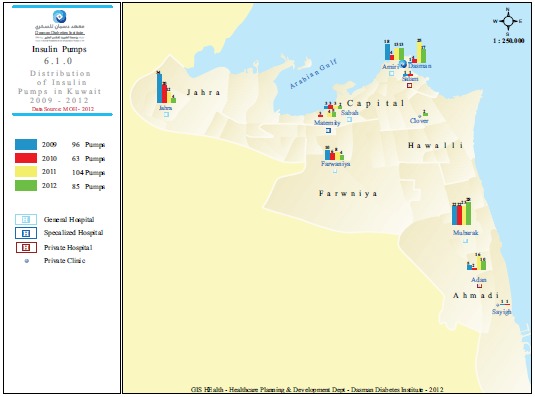
Map showing locations of Healthcare Centres providing insulin pump therapy.

**Table 1:** Glycated haemoglobin (HbA1c) level at baseline and end point for 58 children and adolescents on Continuous Subcutaneous Insulin Infusion (CSII) according to age group.

Age group	n	%	Baseline HbA1c % (mmol/mol)	HbA1c at the end of 12 month observation period % (mmol/mol)	p
< 6 years	3	5.2	8.3 ± 0.6 (67.2)	8.4 ± 0.4 (68.3)	0.815
6-12 years	22	37.9	8.7 ± 1.20 (71.6)	8.1 ±0.8 (65.0)	0.082
>12 years	33	56.9	9.0 ± 1.3 (74.9)	9.5 ± 1.6 (80)	0.363
Total	58	100	8.9 ± 1.20 (73.8)	8.8 ± 1.2 (72.7)	0.701

**Table 2:** Absolute reduction in glycated haemoglobin (HbA1c) from baseline.

Baseline HbA1c (%)	HbA1c Pre-pump	HbA1c at 12 months	Absolute reduction	p
> 9.5	10.3	9.3	0.99	0.004
8.0 – 9.5	8.7	8.5	0.2	0.48
< 8.0	7.5	7.9	-0.43	0.23

**Table 3:** Change in glycated haemoglobin (HbA1c) and absolute reduction or increase in 58 children and adolescents on Continuous Subcutaneous Insulin Infusion (CSII).

Change in HbA1c	n	%
> 1.0 absolute reduction	9	15.52
0.5 – 1.0 absolute reduction	10	17.24
0.1 – 0.4 absolute reduction	8	13.79
No change	11	18.97
0.1- 0.4 absolute increment	6	10.34
0.5 – 1.0 absolute increment	5	8.62
> 1.0 absolute increment	9	15.52
